# Tribological, oxidation and thermal conductivity studies of microwave synthesised molybdenum disulfide (MoS_2_) nanoparticles as nano-additives in diesel based engine oil

**DOI:** 10.1038/s41598-022-16026-4

**Published:** 2022-08-18

**Authors:** Thachnatharen Nagarajan, Mohammad Khalid, Nanthini Sridewi, Priyanka Jagadish, Syed Shahabuddin, Kasturi Muthoosamy, Rashmi Walvekar

**Affiliations:** 1 Faculty of Defence Science and Technology, National Defence University of Malaysia, Kuala Lumpur, Malaysia; 2 Graphene and Advanced 2D Materials Research Group (GAMRG), School of Engineering and Technology, Sunway University, Selangor, Malaysia; 3 Department of Science, School of Technology, Pandit Deendayal Petroleum University, Gandhinagar, Gujarat India; 4 Centre for Nanotechnology and Advanced Materials (CENTAM), Faculty of Engineering, University of Nottingham Malaysia Campus (UMNC), 43500 Semenyih, Selangor Malaysia; 5 Department of Chemical Engineering, School of New Energy and Chemical Engineering, Xiamen University Malaysia, Jalan Sunsuria, Bandar Sunsuria, 43900 Sepang, Selangor Malaysia

**Keywords:** Nanoscale materials, Mechanical engineering

## Abstract

Lubrication has become essential in enhancing engine efficiency in the era of rapid globalising. The tribological, oxidation and thermal conductivity properties of an engine oil play a vital role in improving the quality of a vehicle’s engine life. In this research, molybdenum disulfide (MoS_2_) nanoparticle was synthesised via a microwave hydrothermal reactor. Later, the nanoparticles were dispersed in SAE 20W50 diesel engine oil to formulate the nanolubricant. The results show that nanolubricant with 0.01 wt% MoS_2_ concentration showed the coefficient of friction, average wear scar diameter decreased by 19.24% and 19.52%, respectively, compared to the base oil. Furthermore, the nanolubricant with 0.01 wt% concentration of MoS_2_ nanoparticle showed an enhancement of 61.15% in oxidation induction time in comparison to the base oil. Furthermore, MoS_2_ addition within the base oil demonstrates a ~ 10% improvement in thermal conductivity compared to the base oil.

## Introduction

The automobile industry is well focused on emphasising eco-friendly, quality, durability, and energy efficiency properties. For example, 79% of the fuel is dissipated due to energy loss in a conventional passenger vehicle^1^. Energy loss and mechanical failure are caused mainly by friction and wear. Friction and wear consume around 1/3 of the predominant global energy, and over half of the power accounts for the friction of transportation equipment^2^. Furthermore, worn-out parts account for almost 4/5 of mechanical failure^3^. Friction also contributes to significant issues such as surface corrosion and pollution of the environment. As a result, reducing friction and wear is critical for extending mechanical equipment service life, improving fuel efficiency, and lowering emissions.

Lubrication is one of the most reliable ways to reduce frictional wear, energy saving, environmental protection, and carbon decrement^4^. Many solutions have been used to reduce friction and wear to fulfil energy conservation objectives. Improving the groove texture profile under hydrodynamic lubrication conditions can increase the load-carrying capability of the oil film^5^. On the other hand, their tribological properties are commonly caused by frictional conditions and are prone to wear-out failure after a long service period. Since they may establish a hydrodynamic or elastohydrodynamic lubrication layer on the contact surface during frictional sliding, liquid lubricants are frequently used in the automotive industry^6^. In addition to lubricating oils, ionic liquids can occasionally be used as liquid lubricants^7^. During the beginning and shutdown phases of mechanical parts, or when a high frictional environment occurs, liquid lubricants cannot establish a continuous lubricating layer in the middle of the frictional surfaces. In this context, boundary lubrication and mixed lubrication phases occur, resulting in increased friction and wear. The application of lubricant additives is the prominent method to decrease friction and wear by boundary lubrication^8^. Organic phosphates, organic sulphides, and organic metallic compounds are traditional lubricant additives with strong dispersion stability and tribological qualities. In terms of toxicology, the production of sulphated ash, phosphorous, and sulphur (SAPS), which can produce air contamination such as acid rain and hazy climate^9^ and chemical erosion, are the issues that the environment faces at varying degrees. Although other additives, including ionic liquids, have good tribological properties, their use in the industry is constrained by their high cost and lack of environmental friendliness^10,11^. Nanolubricants use nanoparticles as lubricant additives in the base lubricant, where the particle diameter is usually between 1 and 100 nm^12^. In situ experiments show that incorporating nanolubricants into base oils or coatings reduces friction and wear significantly while also exhibiting intriguing tribological properties. This study aims to improve the tribological qualities of diesel-based engine oil using nano-additives. This is the first attempt to synthesise MoS_2_ nanoparticles using the microwave synthesis route for tribological application. The synthesis of nanoparticles using an advanced microwave synthesis method saves time, energy and produces better tribological, oxidation and thermal conductivity properties than the traditional hydrothermal method^13^. The physicochemical parameters of the MoS_2_ nanoparticles were then determined, and the nanoparticles were dispersed in diesel-based engine oil to develop a novel nanolubricant. Following that, the tribological, oxidation, and thermal characteristics were investigated. The primary goal of this study is to create MoS_2_ nanoparticles using microwave technology, which has improved tribological, oxidation, and thermal properties when dispersed in diesel engine oil. This research will pave the path for developing new microwave-synthesised MoS_2_ nano additives for diesel engine oil.

## Results and discussion

### Characterisation of MoS_2_ nanoparticle and nanolubricant

#### Field emission scanning electron microscope (FESEM) and energy dispersive X-ray spectroscopy (EDS) of MoS_2_ nanoparticle

**Figure 1:**
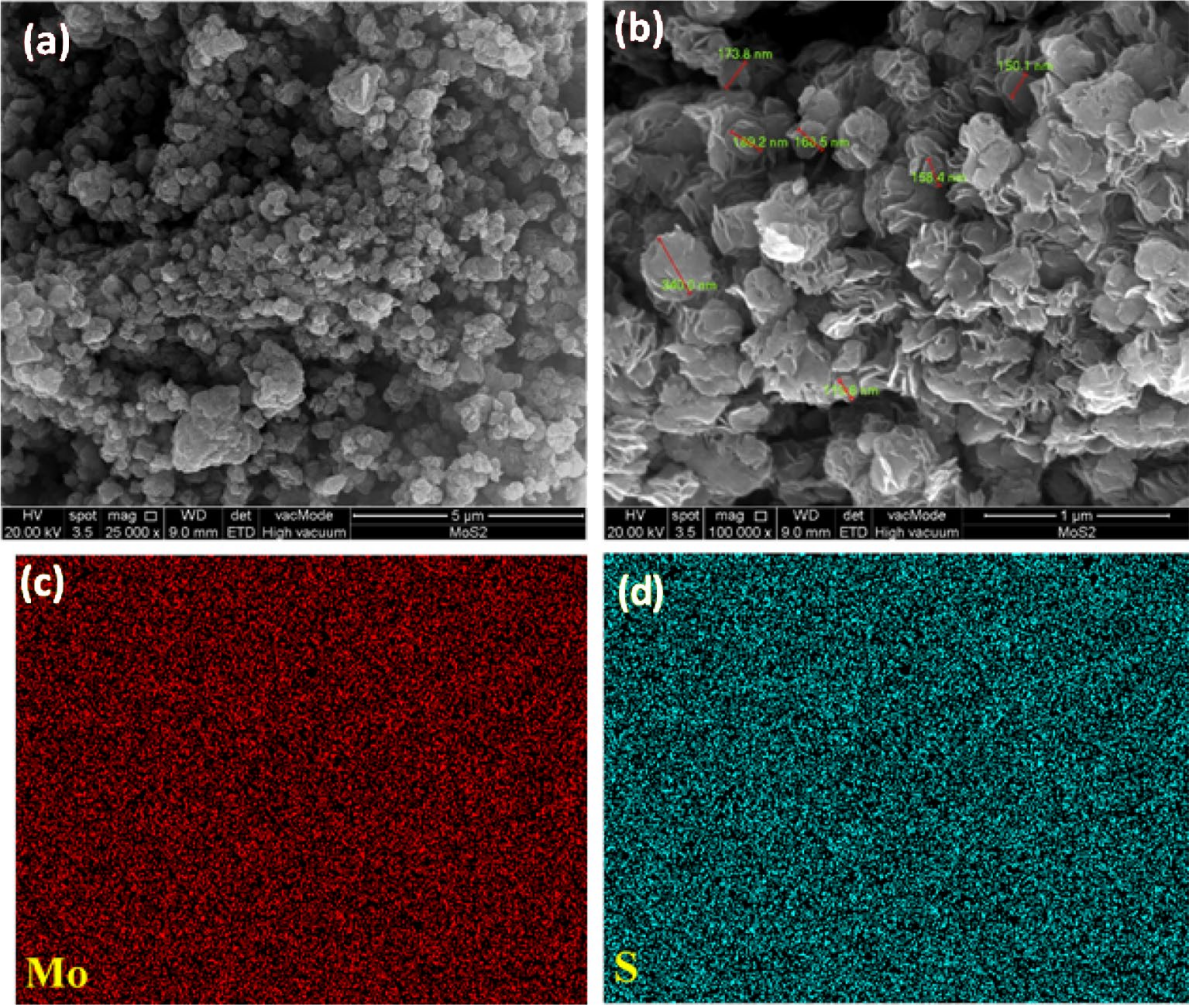
(**a**,**b**) Field Emission Scanning Electron Microscope (FESEM) (**c**,**d**) Energy Dispersive X-Ray Spectroscopy (EDS) mapping of the synthesised MoS_2_ nanoparticle.

**Figure 2:**
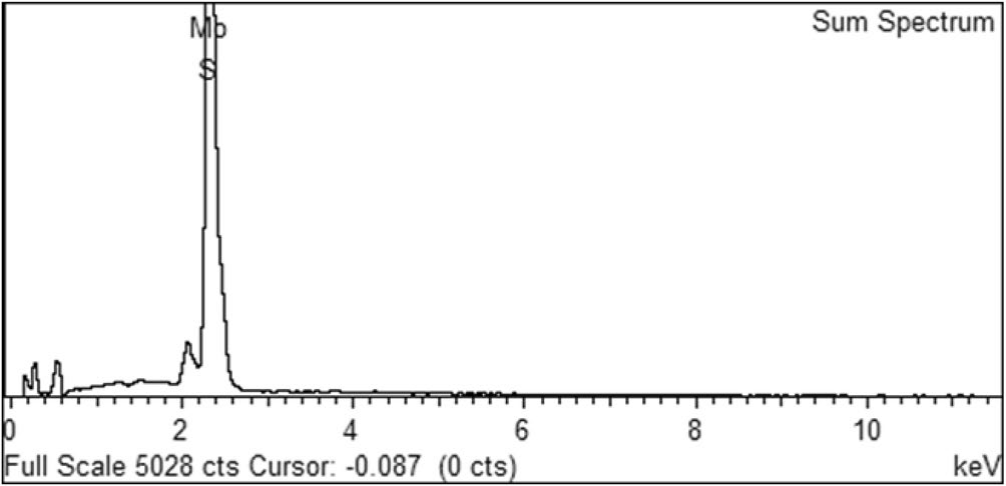
The EDS Spectrum of the MoS_2_ nanoparticles.

Figure 1 shows the morphology of MoS_2_ nanoparticles in (a) 25,000× and (b) 100,000× magnifications. The nanoparticles are uniformly distributed, well-faceted, densely grown, semi-vertically and interleaving lamellar nanosheets with rough edges, confirming the nanosheet morphology of the formed MoS_2_. Figure 1b displays the non-uniformed nanosheets with approximately 150–300 nm sizes. However, several nanosheets are stacked up and seen agglomerated. The uniform and homogeneous distribution of molybdenum and sulfur across the nanosheet are shown in high-resolution EDS elemental mapping in Fig. 1c,d. In addition, the EDS spectrum of the MoS_2_ sample in Fig. 2 confirms the existence of sulfur and molybdenum. The corresponding quantitative surface analysis of EDS in Table 1 presents the elemental distribution of sulfur and molybdenum.

**Table 1:** The elemental distribution of MoS_2_ nanoparticles.

Element	Weight%	Atomic%
S	38.04	64.75
Mo	61.96	35.25
Totals	100.00	

#### X-ray diffraction (XRD) of MoS_2_ nanoparticle

**Figure 3:**
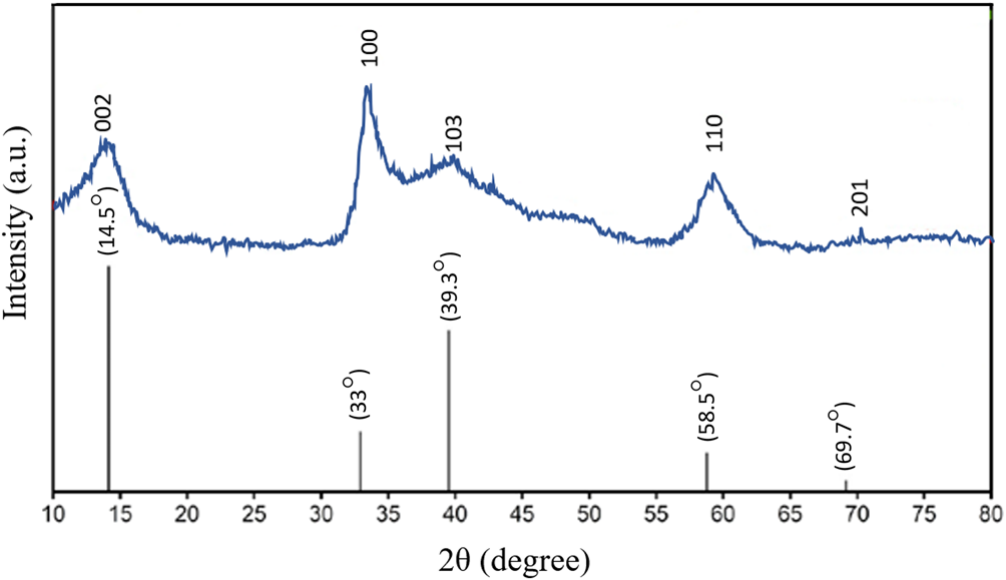
XRD pattern of MoS_2_ nanoparticles.

Figure 3 shows the XRD diffraction peaks of MoS_2_ at 2 = 14.5°, 33.0°, 39.3°, 58.5°, and 69.7°, which can be referred to as the (002), (100), (103), (110), and (201) peaks of pure hexagonal MoS_2_ phase according to JCPDS card no.371492, which are in accordance with previous studies^14,15^. Peak broadening implies that the crystalline size is very small. For (100) and (103) XRD peaks, the magnitude dissimilarity between the reference pattern in the JCPD card and the synthesised nanoparticle is due to differences in texture of crystallite size difference and the size of the scattering domains. The crystallite size is estimated by using the Scherrer Eq. (1)

1 $$D = \frac{K\lambda }{{\beta \cos \theta }}$$

where D is the crystallite size (nm), K = 0.9 (Scherrer constant), λ is the wavelength of X-rays, β is the full width at half maximum (FWHM), and θ represents the peak position.

According to Eq. (1), the crystallite size of the MoS_2_ nanoparticles was 53.6 nm. Furthermore, other peaks of separate phases or impurities are not found in the XRD patterns, indicating that the crystal structure of MoS_2_ nanosheets is of high purity.

#### Fourier-transform infrared spectroscopy (FTIR) of MoS_2_ nanoparticle

**Figure 4:**
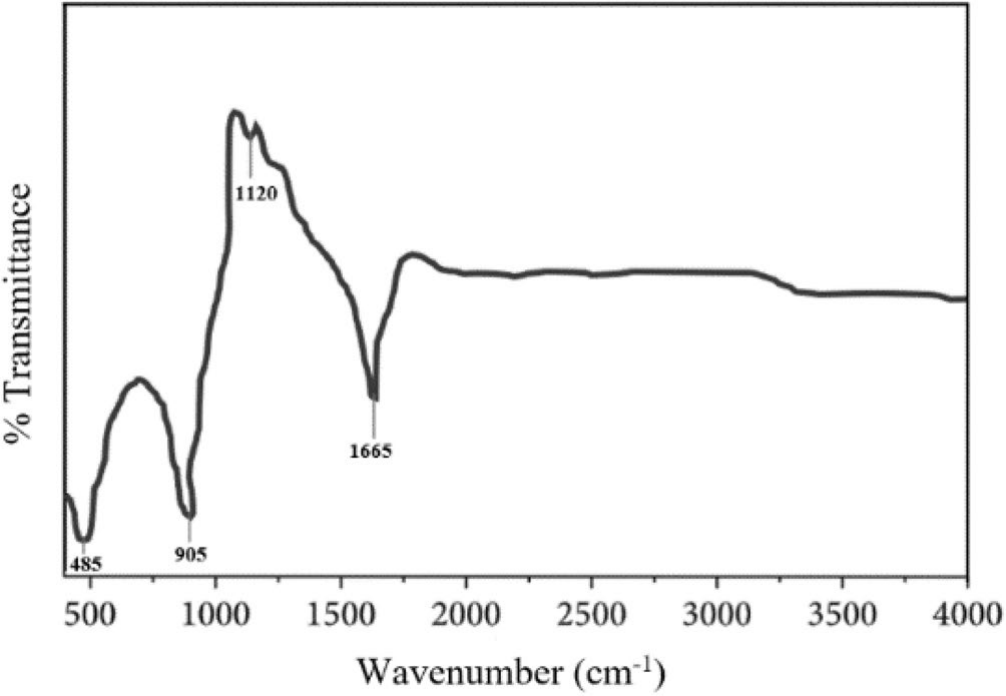
FTIR spectroscopy graph of MoS_2_ nanoparticle.

Figure 4 displays the FTIR spectra of the MoS_2_ nanoparticle. The peaks were confirmed using the FTIR application library and the journals. Both samples have strong absorption bands at 485 cm^−1^, 905 cm^−1^, 1120 cm^−1^, and 1665 cm^−1^. The Mo-S bond is responsible for the band at 485 cm^−1^, while the S–S bond is responsible for the band at 905 cm^−1^. The stretching vibrations of the hydroxyl group and Mo–O vibrations are responsible for the absorption band between 1120 and 1665 cm^−1^^16^. By revealing the functional groups present in the study, the FTIR spectra further confirm the formation of MoS_2_.

#### Visual observation and zeta potential of MoS_2_ nanolubricant

**Figure 5:**
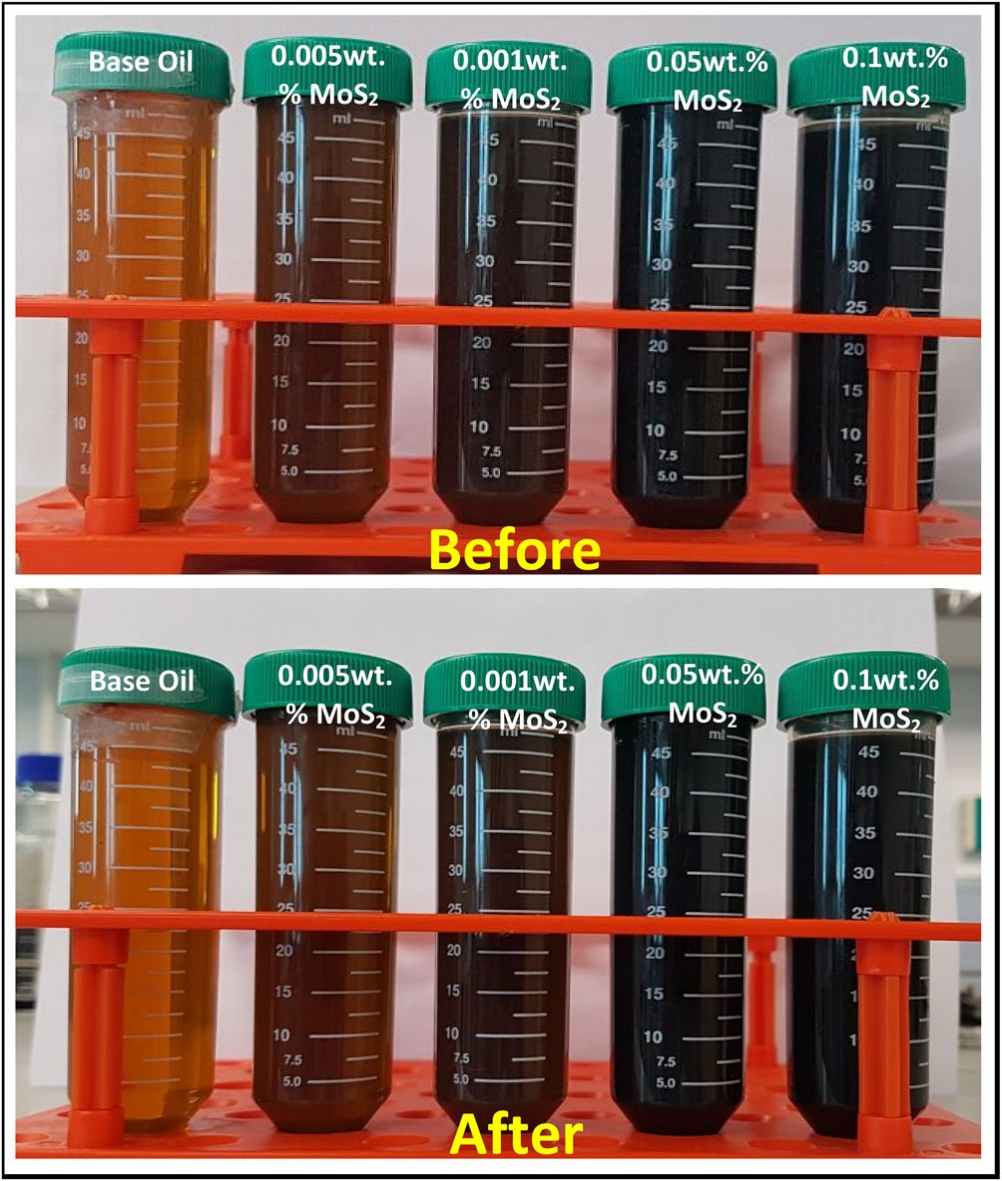
Visual observation of the dispersion stability of MoS_2_ nanolubricants at varying concentrations.

The stability of the MoS_2_ nanolubricants against sedimentation via visual observation showed that the four various concentrations of 0.1 wt%, 0.05 wt%, 0.01 wt%, and 0.005 wt% of MoS_2_ based nanolubricants were stable against sedimentation for 21 days (Fig. 5). The zeta potential is significant as its magnitude is used to determine the stability of colloidal dispersions. As shown in Table 2, the zeta potential value of the MoS_2_ nanolubricant with 0.05 wt%, 0.01 wt% and 0.005 wt% of MoS_2_ concentrations is higher than 60 mV, indicating the nature of MoS_2_ nanoparticles to be extremely stable in the nanolubricant. While 0.1 wt% shown lower zeta potential value, indicating poor stability in the engine oil as the nanoparticle concentration is the highest.

**Table 2:** Zeta potential magnitude of the MoS_2_ nanolubricant with different concentrations.

MoS_2_ concentration in nanolubricant (wt%)	Zeta potential (mV)
0.005	372.8
0.01	279.7
0.05	158
0.1	37

Dispersion with a higher zeta potential (negative or positive) is electrically stable, whereas those with a lower zeta potential agglomerate or flocculate. In general, the arbitrary value of 25 mV (positive or negative) distinguishes low-charged exterior from a highly charged exterior. Dispersion with a zeta potential of 40 to 60 mV is considered fairly consistent, whereas those with more than 60 mV are considered extremely stable. The value of the zeta potential is directly proportional to the dispersion stability of the materials^17^.

### Tribological analysis of MoS_2_ nanolubricant

**Figure 6:**
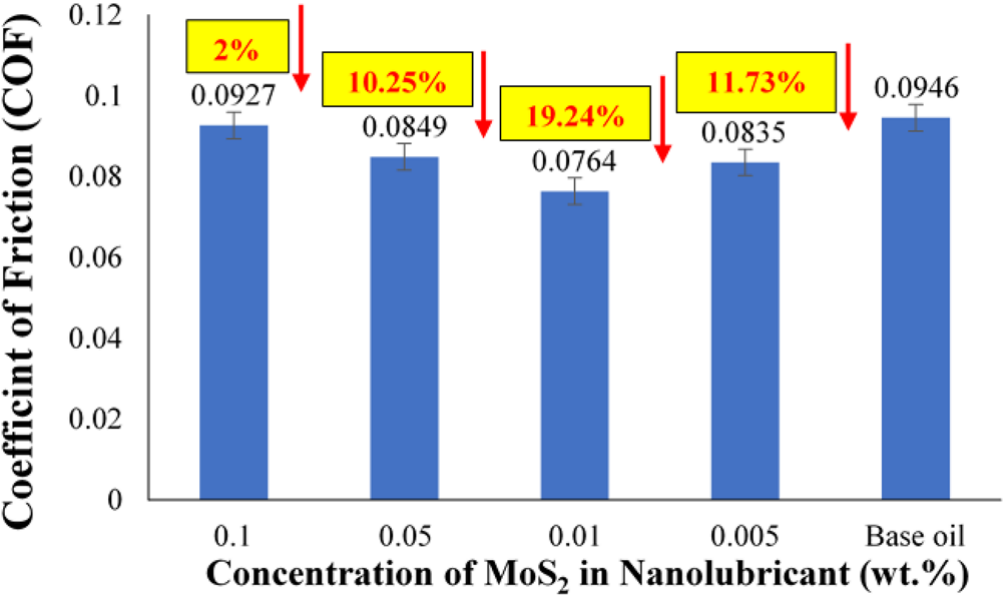
COF of MoS_2_ nanolubricant.

The coefficient of friction of MoS_2_ nanolubricant with varying concentrations of MoS_2_ nanoparticle wt% in the base oil is shown in Fig. 6. Without any nano-additives, the friction coefficient of the base oil was 0.0946. The friction coefficient of base oil with MoS_2_ nanoparticles was found to be lower than pure base oil. In comparison to the base oil, the COF was reduced to 2%, 10.25%, 19.24%, and 11.73% for 0.1 wt%, 0.05 wt%, 0.01 wt%, and 0.005 wt%, respectively. When the MoS_2_ percentage in the nanolubricant was increased from 0.01 wt%, some MoS_2_ nanoparticles agglomerate, resulting in larger secondary particle size. As a result, friction and wear would worsen, resulting in an increase in COF. The lowest concentration of MoS_2_ nanoparticles, 0.005 wt% was insufficient to cover the entire contact surface, resulting in a greater COF than 0.01 wt% MoS_2_. This suggests that 0.01 wt% of MoS_2_ nanolubricant is the best concentration for reducing COF. The sliding of nanosheets causes this phenomenon at asperities and deformed surfaces of individual nanosheets at interfaces to produce a protective layer known as the tribofilm, which decreases the COF^18–20^. The development of a tribofilm comprising nanosheets aids in reducing the friction caused by the individual layers of nanosheets slipping.

The findings show some damage caused by adhesive wear under the applied stress due to continuous sliding friction. Because of their higher surface energy and many dangling S bonds, MoS_2_ nanoparticles can readily react and produce an abrasion-resistant protective coating at contacting surfaces. The MoS_2_ nanosheets will be captivated into frictional surfaces, generating an adsorbed film and forming S–O or S–Fe bonds. The oxide layer on the substrate's surfaces provided the O and Fe. The adsorbed coating eliminated direct contact between frictional contacts and increased tribological characteristics^21^. The effect of firm boundary lubrication between the frictional pairs develops a protective tribofilm. Due to the adequate lubricity, this may result in an excellent ability to withstand shear failure.

**Figure 7:**
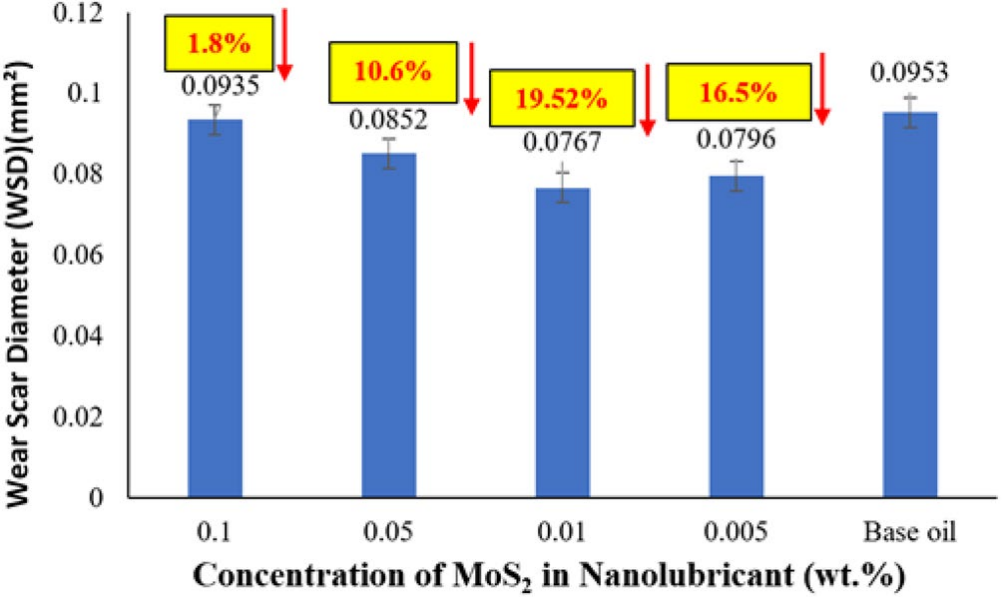
Average WSD profile on MoS_2_ nanolubricant.

**Figure 8:**
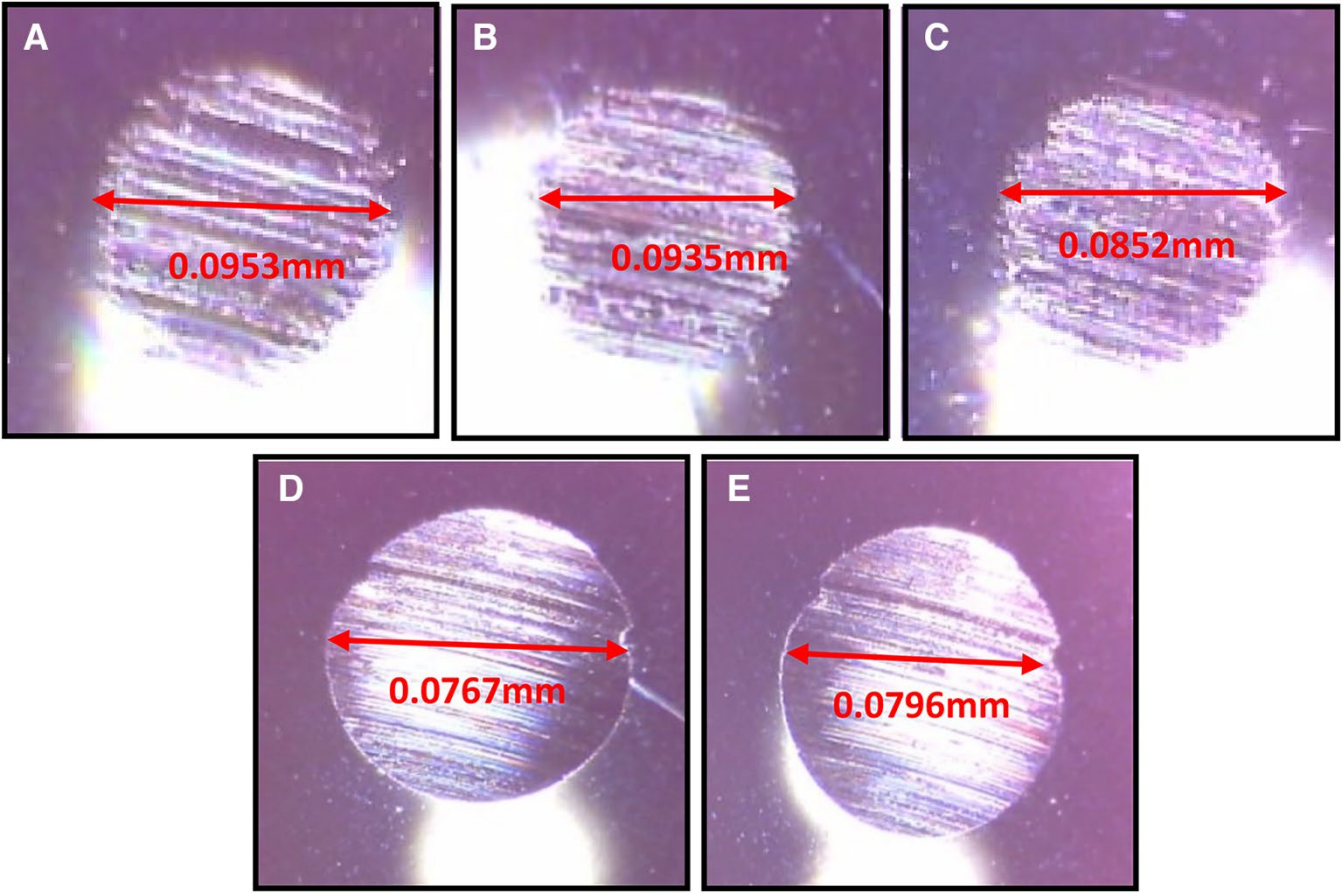
Images of steel balls after tribological analysis using base oil (**A**), 0.1 wt% MoS_2_ (**B**) and 0.05 wt% MoS_2_ (**C**) 0.01 wt% MoS_2_ (**D**) and 0.005 wt% MoS_2_ (**E**).

Figure 7 presents wear scar diameter details of MoS_2_ nanolubricant with various MoS_2_ nanoparticle wt.% in base oil concentrations. The image of the wear scar diameter created on the steel balls during the tribological trials is depicted in Fig. 8. When the four-ball test was conducted for the tribological study, the WSD for the base oil without nanoparticle addition was 0.0953. However, adding MoS_2_ nanoparticles to the base oil minimises the WSD. In comparison to the base oil, the WSD is reduced by 1.8%, 10.6%, 19.52%, and 16.5% for 0.1 wt%, 0.05 wt%, 0.01 wt%, and 0.005 wt%, respectively. This demonstrates that 0.01wt.% MoS_2_ produces the lowest WSD in tribological analysis. In Fig. 8, the wear scar images of base oil (A), nanolubricant with 0.1 wt% (B) and 0.05 wt% (C) MoS_2_ exhibited darker concentric grooves, indicating abrasive wear, but smaller percentages of MoS_2_, such as 0.01 wt% (D) and 0.005 wt% (E) exhibited smoother wear tracks, indicating decreased contact surfaces between the steel balls. The darker furrow is deeper, whereas the brighter furrow is shallower. Suresha et al.^22^ made a similar observation. These ridges are responsible for depositing the MoS_2_ nanoparticles firmly on the wear surface, which results in a reduction in wear. Huang et al. reported a similar process with graphite sheets^23^. In another experiment, Hernandez et al. demonstrated that nanoparticles aggregate in the wear scar region^24^. In comparison to the base oil containing MoS_2_ nanoparticles, the wear scar image of the steel ball lubricated by the base oil displayed many broad and deep ridges. This might be due to the many MoS_2_ nanosheets penetrating more easily into the lubricant contact. Furthermore, nanosheets can create a continuous layer on rubbing surfaces because of their excellent contact adherence, improving tribological qualities. This phenomenon is known as the mending effect, where MoS_2_ nanoparticles settle and occupy the grooves on the worn surface scratches of the rubbing surfaces, avoiding direct contact between the two surfaces and lowering the WSD. Those described above experimental tribological results imply that with an ideal concentration of 0.01wt% MoS_2_ in the engine oil, both COF and WSD can be significantly improved.

According to the initial study, the formation of tribofilm and the mending effect is the fundamental mechanism for decreasing the frictional wear in the case of MoS_2_ based nanolubricant. Due to the planar geometry of MoS_2_, it may readily slide between the oil's surfaces. Furthermore, MoS_2_ will cluster or agglomerate together and precipitate as the concentration increases, increasing wear and friction between surfaces. The segregation of interlayers into distinct layers is attributed to the wear process of MoS_2_ nanosheets due to weaker van der Waals or Coulombic repulsive interactions at contact compulsion^25,26^. These findings indicate that adding MoS_2_ to the lubricant significantly improves the nanolubricant's tribological capabilities.

### Oxidation analysis of MoS_2_ nanolubricant

**Figure 9:**
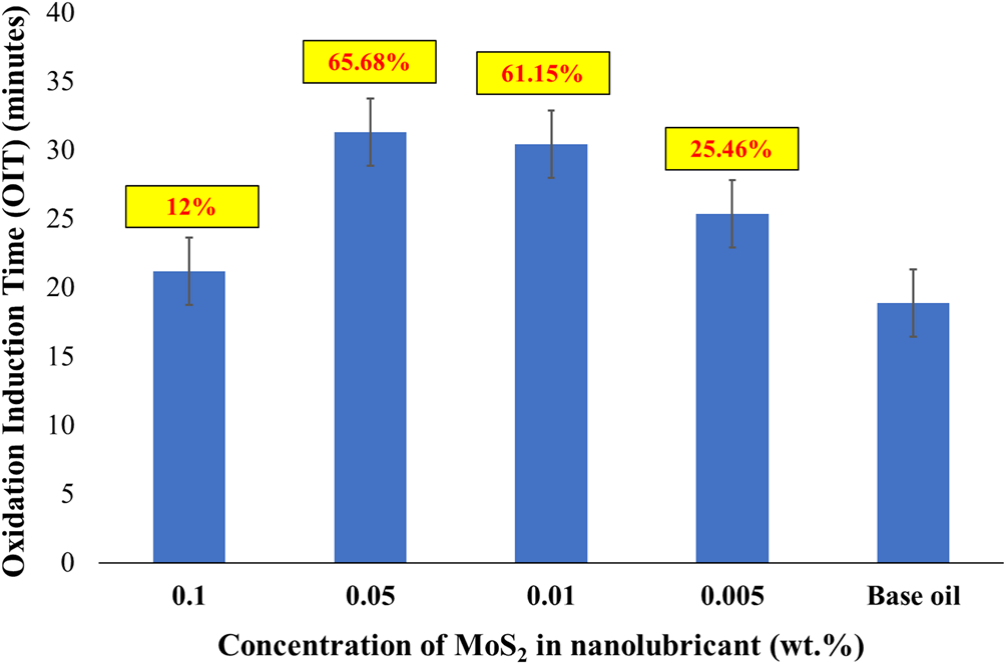
OIT of MoS_2_ nanolubricant with different concentrations.

In automobile industry, lubricants endure oxidation caused by intense temperature, high load, and continuous contact with air. Oxidation accelerates the degradation process of the base oils and additives, which decreases their performance, efficiency and lifespan. The results of the OIT of the nanolubricants are shown in Fig. 9. Compared to the base oil, the OIT was improved by 12.17%, 65.68%, 61.15% and 25.46% for 0.1 wt%, 0.05 wt%, 0.01 wt%, and 0.005 wt%, respectively. The nanolubricant with 0.05 wt% of MoS_2_ nanoparticles showed the highest OIT compared with other concentrations of nanolubricant formulation.

Due to the synergistic effect of MoS_2_ with Zinc-dialkyl dithiophosphate (ZDDP), the nanolubricant's antioxidant characteristics were increased. The ZDDP additive is one of the most extensively employed additives in the automotive sector. It is most recognised for its antiwear characteristics, but it also contains antioxidant and extreme pressure characteristics. Phosphate capacity to digest oxides appears to be linked to ZDDP antiwear capabilities. Several authors^27–29^ have demonstrated a synergistic interaction between MoDTC and ZDDP due to MoS_2_ production. Lubricants undergo a three-step oxidation process. A free radical is produced at the first stage, initiation. The free radical combines with oxygen to produce peroxide radicals in the second stage called propagation. After combination with other lubricant components, these radicals have additional radicals. In the third phase, two radicals join to form a stable molecule, known as the termination stage. The synergistic effect of MoS_2_ with ZDDP promotes hydrogen donation, which stops the radical propagation process. It causes the nanolubricant’s OIT to be higher. The nanolubricant with 0.1wt.%, 0.01wt.% and 0.005wt.% of MoS_2_ nanoparticles possesses lower OIT than 0.05wt.% and 0.01wt.% as the mentioned concentration are not optimum in provide higher OIT in the nanolubricant. The substantial improvement in OIT of nanolubricants shows that the synergistic effect of MoS_2_ nanoparticles and ZDDP can exhibit good oxidation stability, enhancing the antioxidant properties of the nanolubricants.

### Thermal conductivity analysis

**Figure 10:**
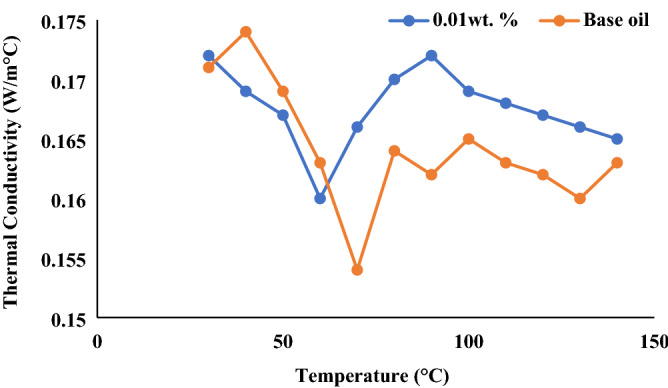
Thermal conductivity of 0.01 wt% nanolubricant with base oil.

From the tribological and oxidation analysis, the nanolubricant with 0.01 wt% MoS_2_ nanoparticles provide good results compared to other concentrations of MoS_2_ nanoparticles in nanolubricant. Thus, this concentration was further investigated for its thermal conductivity using the laser flash method. The addition of MoS_2_ within the base oil demonstrates an improvement in thermal conductivity, as shown in Fig. 10. The thermal conductivity of the nanolubricant showed approximately ~ 10% improvement compared to the base oil. Due to the lower concentration of MoS_2_ nanoparticles (0.01 wt%), the notable improvement in thermal conductivity was caused by the molecular collisions among the base oil and nanoparticles^30–34^. Moreover, perceived thermal conductivity behaviour during the investigation indicates that this enhancement is due to the percolation mechanism and the involvement of the Brownian motion of the nanosheet^35–37^. In addition, the nanoparticles' phonons get scattered in the active nanostructures, improving the contact conductance^38^.

Subsequently, thermal conduction channels are developed, which improves thermal conductivity. This scenario is known as the percolation mechanism. In addition, the thermal transfer among the colliding nanoparticle rose the thermal conductivity of the nanolubricant. For example, from Fig. 10, the thermal conductivity of the nanolubricant increases more than the base oil after the temperature of 60 °C as a more intense Brownian motion of the nanoparticles occurs^39^. This thermal transport phenomenon in the nanolubricant was due to the physio-chemical attribute of the base oil, as well as collaboration with the reinforcement nanoparticles.

## Conclusion

In the tribological analysis, nanolubricant with 0.01 wt% concentration of MoS_2_ nanoparticle shows the best results in reducing friction coefficient and wear scar diameter with 19.24% and 19.52% decrement compared to the COF of the base oil. It is due to the formation of protective film known as the tribofilm, which forms in between the frictional surfaces, significantly reducing the COF. The reduction in WSD was caused by the phenomenon known as the mending effect, where the MoS_2_ nanoparticles settle and fill the furrows on the worn surface scratches of the rubbing surfaces, avoiding direct contact between the two surfaces and lowering the WSD. In the OIT analysis, nanolubricant with 0.01wt.% concentration of MoS_2_ nanoparticle shows the best results with 65.68% enhancement in OIT compared to the base oil. MoS_2_ nanoparticles can exhibit good oxidation stability, enhancing the antioxidant properties of the nanolubricants. Therefore, incorporating the MoS_2_ nanoparticles improves the performance of the nanofluids significantly, as the nanolubricant with 0.01 wt% MoS_2_ nanoparticles concentration provides the best result in COF, WSD and OIT compared to other concentrations. Further, the addition of MoS_2_ demonstrated an improvement trend in thermal conductivity with ~ 10% enhancement compared to the base oil. This is due to the percolation mechanism, which may increase the thermal conductivity. All tests confirm that 0.01wt% MoS_2_ based nanolubricant showed the highest enhancement in tribological, oxidation and thermal conductivity analysis.

## Methods

### Materials

All chemical substances utilised in the experiment were of analytical grade and used as received without further purification. Chemicals such as ammonium molybdate tetrahydrate ((NH_4_)6Mo_7_O_24_.4H_2_O) (Fisher Chemicals- Chicago, USA), thiourea (SC(NH_2_)_2_) (R&M Chemicals- Dundee, UK) and ethanol (CH_3_CH_2_OH) (Sigma-Aldrich, USA) were used for MoS_2_ nanoparticle synthesis. The lubricant oil used was diesel engine oil with API SAE 20W50 CD/SE GL-4.

### Preparation of MoS_2_ nanoparticles using advanced microwave synthesis

**Figure 11:**
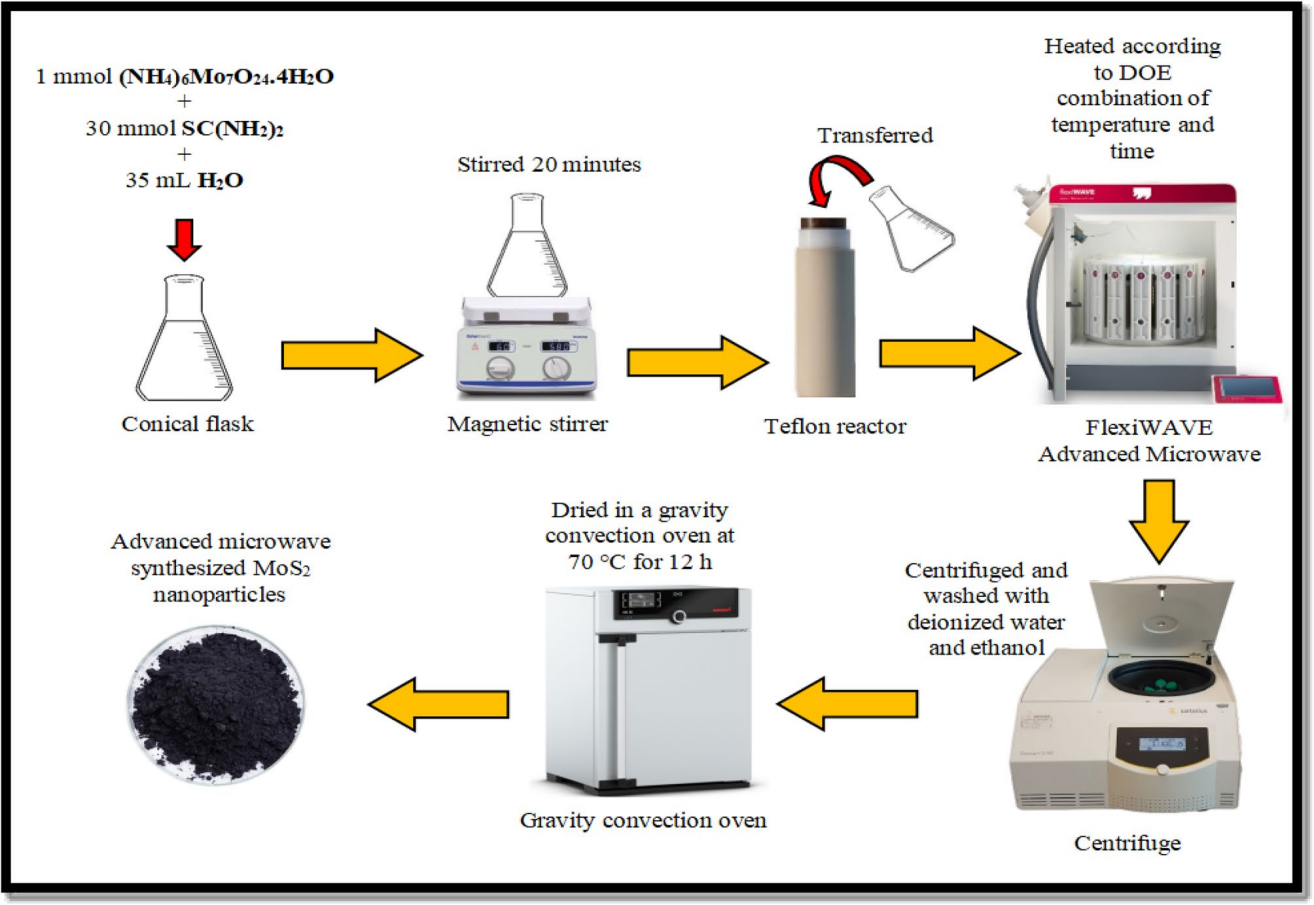
Schematic diagram of MoS_2_ nanoparticles preparation via advanced microwave synthesis.

To synthesise MoS_2_ nanoparticles, all precursors were stoichiometrically measured and calculated as accurately as possible using a Sartorius PRACTUM224-1S analytical balance with a precision of ± 0.1 mg. Later, in 35 mL of deionised water, one mmol ammonium molybdate tetrahydrate ((NH_4_)6Mo_7_O_24_.4H_2_O) and 30 mmol thiourea (SC(NH_2_)_2_) were dissolved. The solution was stirred at 700 RPM for 20 min at room temperature with a Fisherbrand™ Isotemp™ hot plate stirrer. For synthesis reaction, a microwave platform system (Milestone flexiWAVE) was used. The homogeneous solution was transferred into a Teflon vessel of the microwave synthesis platform. The solution was heated to 200 °C for 15 min. After the synthesis ended, the reaction mixture was left to cool naturally to room temperature ( ∼26 °C). The samples were then centrifuged using Sartorius Centrisart® D-16C (Göttingen, Germany) universal benchtop centrifuge with a maximum speed of 9000 min^−1^. The samples were washed several times with deionised water before being soaked in ethanol. The samples were then dried for 12 h in a Memmert UN55 gravity convection oven (Schwabach, Germany) at 70 °C. The powder was then smoothly ground using a 50 mm natural Agate mortar and pestle. Figure 11 illustrates the overall process involved in synthesising the MoS_2_ nanoparticles.

### MoS_2_ based nanolubricant formulation

**Figure 12:**
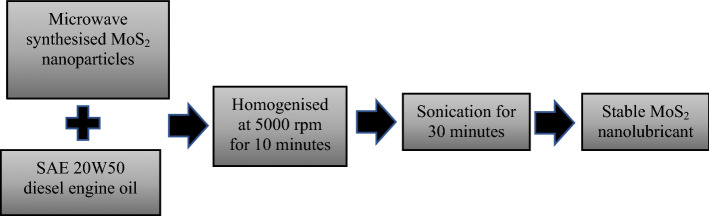
Process flow of MoS_2_ nanolubricant formulation.

To synthesise the nanolubricant, 0.1 wt%, 0.05 wt%, 0.01 wt%, and 0.005 wt% of the collected MoS_2_ nanoparticles were dispersed separately in 100 ml of SAE 20W50 diesel engine oil. The mixture was then homogenised for 10 min at 5000 RPM using a Silverson L5M-A high shear lab mixer. The samples were sonicated further for 30 min in Cole-Parmer ultrasonic bath (Vernon Hills, USA) to enhance stability and ensure that all nanoparticles were uniformly mixed in the base oil without agglomeration. The formulated nanolubricants were highly stable for more than one week. Figure 12 depicts the overall process of nanolubricant formulation. The MoS_2_ nanolubricants with various concentrations were prepared and stored in a sealed container under room temp for further analysis of their application, such as tribology, oxidation induction time and thermal conductivity.

### Physio-chemical characterisation of MoS_2_ nanoparticles and nanolubricant

MoS_2_ nanoparticles were characterised physicochemically by Field Emission Scanning Electron Microscopy (FESEM), Energy-Dispersive X-ray spectroscopy (EDS), X-ray Diffractometer (XRD) and Fourier-Transform Infrared Spectroscopy (FTIR). In addition, MoS_2_ nanolubricant were characterised by Zeta potential and visual observation. All physio-chemical characterisation of MoS_2_ nanoparticles and nanolubricantas are as described follows:

#### Field emission scanning electron microscopy (FESEM) and energy-dispersive X-ray spectroscopy (EDS)

The size and morphology of MoS_2_ nanoparticles were studied using an FEI Quanta 400F, USA, by attaching the samples on stubs with conductive carbon tape. The machine was run at a high vacuum of 20 kV. To obtain samples' morphological details, different magnifications from 25,000 × to 100,000 × were used. In addition, the elemental compositions of MoS_2_ nanoparticles were also assessed using Energy-Dispersive X-ray spectroscopy (EDS).

#### X-ray diffractometer (XRD)

PANalytical X-ray Diffractometer was used to collect XRD data. A sample of MoS_2_ nanoparticles was scanned from 20 to 80 degrees at a step size of 1 degree/min. The size of the divergence slit is 0.9570 degrees. Copper material was used to generate X-rays with a wavelength (K alpha) of 1.54 angstroms. X-rays were filtered through Ni using an operational voltage of 45 kV and a current of 27 mA.

#### Fourier-Transform Infrared Spectroscopy (FTIR)

Fourier-Transform Infrared Spectroscopy (FTIR), Spectrum Two™ Perkin Elmer (L160000M), was employed to identify the functional groups of MoS_2_ nanoparticles. The spectra were obtained from spectral wavenumber of 500 to 4000–1 with 200 scans.

#### Zeta potential

The zeta potential of suspensions was determined using the Zetasizer Nano (Malvern, Worcestershire, UK) to determine their stability. Between the particle surface and the dispersion liquid, there is an electric potential at the sliding plane. The device combines electrophoresis and laser Doppler velocimetry, which detects a particle's velocity in a liquid when electrical energy is exerted. Because the oil's viscosity index and dielectric constant are known, which is 115 and 2.4, the Henry equation uses the Smoluchowski equation to compute the Zeta potential.

#### Visual observation of nanolubricant

The stability of the MoS_2_ nanolubricants against sedimentation was studied by visual observation. The samples in centrifuge tubes were visually monitored for stability against sedimentation for 21 days.

### Evaluation of tribological properties of MoS_2_ nanolubricant

A Ducom four-ball tribotester TR-30L was used to assess the COF and average WSD of MoS_2_ nanolubricants with concentrations of 0.1 wt%, 0.05 wt%, 0.01 wt%, and 0.005 wt%, as well as the base oil. Steel balls were immersed in nanolubricants for tribological testing where a Steel ball rotates in contact with another three metal balls in a ball plot. The diameter of the steel ball used in the test was 12.7 mm. The physical parameters of the steel ball used are listed in Table 3. In order to avoid contamination, the steel balls and associated apparatus were washed in ethanol and dried before tribological experiments. The rotational speed, applied load, time, and temperature were 12,000 rpm, 392.5 N, 3600 s, and 75 °C, according to the ASTM 4172–94 standard. Under the frictional contact of the four metal balls, the ASTM 4172–94 standard conditions aid in the early examination of the lubricant's antiwear qualities. Table 4 lists the operational parameters of a four-ball tribotester. The COF of the nanolubricant was determined by the main data processor on the tribotester. The diameter of the wear scar was evaluated by the image acquisition device. After the four-ball test, the worn scar diameter of the fixed metal balls is measured to ascertain the extent of wear. Throughout the experiment, the lubricant was maintained at a consistent temperature of 75 °C. The steel balls were washed with ethanol, and the worn scar was studied with an optical microscope. The coefficient of friction was measured using Eq. (2)

2 $$\mu = 2.22707 \frac{\tau }{p}$$

where μ is the coefficient of friction for the experimental samples, the average frictional torque, τ in kg-cm and p, is the load exerted while carrying out the investigation.

**Table 3:** Physical properties of the steel ball.

Properties	
Material	Carbon-chromium steel
Hardness (*H*), HRC	1
Density (ρ), gm/cm^3^	7.79
Surface roughness (Ra),μm	0.022

**Table 4:** Operating Parameters for four-ball tribotester.

Parameter	Purpose
Rotating speed	12,000 rpm
Load applied	392.5 N
Time	3600 s
Temperature	75 °C

### Evaluation of the oxidation induction time (OIT) properties of MoS_2_ nanolubricant

The OIT of MoS_2_ nanolubricants was determined using pressure-DSC at four various concentrations: 0.1 wt%, 0.05 wt%, 0.01 wt%, 0.005 wt%, and the base oil. These tests were achieved with the TA instrument's High-Pressure Differential Scanning Calorimeter (HP-DSC) 25P. The use of HP-DSC to study the oxidative stability of nanolubricants necessitates an airtight sample chamber. HP-DSC measures temperature flow for pressure-sensitive substances by attributing the heat flow of a blank reference pan to a sample pan. This procedure was carried out under accelerated conditions of 500 psi pressure and 200 °C isothermal temperature. For all experiments, approximately 3.2 mg of nanolubricant was placed into the HP-DSC, and the samples were first allowed to equilibrate at 50 °C. Table 5 lists the operating parameters of the P-DSC. The relationship between the kinetic rate constant (k) and the temperature (T) in kinetic expressions, such as those governing OIT measurements, is given by the Arrhenius expression in (3)

3 $$k\left( T \right) = Z_{\exp }^{{\frac{ - E}{{RT}}}}$$

where k(T) is the specific rate constant at temperature T (1/min), Z represents the pre-exponential factor (1/min), E represents the activation energy (J/mol), R is the molar gas constant (8.3143 J/mol K), and T represents absolute temperature (K).

**Table 5:** Operating condition for P-DSC.

Parameter	Purpose
Sample Amount	3 ± 0.3 mg
Gas	Ultra-high purity (UHP) grade oxygen
Flow rate	50 ml/min
Temperature	200 °C
Pressure	500 psi
Ramp Rate	10 °C/min

### Evaluation of the thermal conductivity properties of MoS_2_ nanolubricant

The samples were first injected into the sample ring with a syringe. The sample was then filled in the sample ring, which was critical for homogeneous irradiation. Finally, the upper and lower sealing discs were sprayed with graphite before assembling the sample holder's components to promote black body absorption. The heating was applied from room temperature to 140 °C at a rate of 10 °C/min. The chamber's atmosphere is nitrogen. The operating parameters of the NETZSCH 467 HT HyperFlash® are shown in Table 6.

**Table 6:** Operating condition for LFA.

Parameter	Purpose
Gas	Ultra-high purity (UHP) grade Nitrogen
Heating rate	10 °C/min
Temperature	Room temperature to 150 °C

## Data Availability

All data generated or analysed during this study are included in this published article. Additional data is available from the corresponding author on request.
